# Torque-Dependent Anchor Loss and Fourth-Harmonic Damping Anisotropy in Coriolis Vibratory Gyroscopes

**DOI:** 10.3390/s26082483

**Published:** 2026-04-17

**Authors:** Ning Wang, Zhennan Wei, Guoxing Yi, Yanyu Sun, Changhong Wang

**Affiliations:** Department of Control Science and Engineering, Harbin Institute of Technology, Harbin 150001, China; ning.w@stu.hit.edu.cn (N.W.); ygx@hit.edu.cn (G.Y.); 20200155@hit.edu.cn (Y.S.); cwang@hit.edu.cn (C.W.)

**Keywords:** Coriolis vibratory gyroscope, hemispherical resonator gyroscope, anchor loss, partial-slip friction, elastic-wave radiation, quality factor, damping anisotropy

## Abstract

The quality factor (*Q*) and its circumferential non-uniformity are essential for the resolution and long-term stability of Coriolis vibratory gyroscopes (CVGs). In practice, packaging and mounting anchors introduce torque-dependent and circumferentially non-uniform anchor dissipation, resulting in harmonic damping anisotropy. This paper presents an energy-consistent framework that quantitatively relates the tightening torque to both the mean damping factor η=1/Q and its circumferential harmonic components. A hemispherical resonator gyroscope (HRG) is used for validation, where the dominant component is the fourth harmonic. By decomposing the energy dissipated per cycle, anchor loss is separated into friction loss and radiation loss. The friction channel is modeled using a partial-slip contact energy loss formulation combined with an equivalent tangential impedance coupling description, leading to a torque power-law scaling suitable for parameter identification. The radiation channel is described by an impedance coupling model that captures torque-enhanced anchor stiffness and potential saturation leakage under strong coupling. Controlled torque experiments show that η(ϑ) exhibits an almost pure fourth-harmonic dependence on the standing wave orientation for all tested torques. Within the accessible torque range, the mean damping decreases slightly with torque, while the harmonic amplitude increases and the phase progressively converges, supporting a friction-dominated interpretation. The phase convergence further suggests progressive stabilization of the contact state. The proposed approach provides quantitative guidance for torque selection and anchor structure design in resonant gyroscopes.

## 1. Introduction

Coriolis vibratory gyroscopes (CVGs) have become core inertial devices for aerospace, defense navigation, and deep-space exploration, owing to their high accuracy, long service life, and strong immunity to external disturbances [1,2,3]. In parallel, miniaturized and low-power CVGs are being increasingly adopted in civilian scenarios such as autonomous driving, unmanned aerial vehicle (UAV) control, and industrial robotics, where they enable autonomous navigation and high-precision motion control [4,5,6,7]. A key parameter that fundamentally limits CVG performance is the resonator quality factor (*Q*) [8]. The quality factor quantifies damping as the ratio of the energy stored in the resonator to the energy dissipated per oscillation cycle, and thus directly governs the decay of resonant motion. A higher *Q* indicates lower dissipation, leading to improved sensitivity, a more favorable bandwidth–noise trade-off, and ultimately higher resolution, accuracy, and long-term stability [9,10]. For convenience, dissipation is often expressed by the damping factor η=1/Q, where a smaller η corresponds to lower energy loss and a higher *Q*. In addition to the absolute *Q* level, its circumferential uniformity is equally important. Damping anisotropy breaks the symmetry of resonant modes, aggravates mode matching errors, increases the control burden for suppressing quadrature errors, and degrades device-to-device consistency and long-term stability [11,12,13,14]. Therefore, improving both the magnitude and the circumferential uniformity of *Q* remains one of the critical challenges for high-performance CVGs.

The quality factor *Q* of CVG resonators is jointly determined by multiple dissipation mechanisms, which are commonly attributed to material-related internal losses, gas damping, and anchor-related losses [15,16,17]. Material-related losses include bulk internal friction, surface/subsurface damage-induced dissipation, and thermoelastic damping [18,19,20]. Among them, surface and subsurface damage are closely related to fabrication processes and surface integrity, and may become a dominant limitation to *Q* under high-vacuum conditions where external damping is largely suppressed [19,21]. Gas damping mainly arises from molecular collisions and viscous shear in the package cavity, and can typically be reduced substantially via high-vacuum packaging. As material loss and gas damping are progressively minimized, anchor-related dissipation introduced by packaging and mounting often becomes the dominant term in the residual loss budget. More importantly, it can induce circumferential damping anisotropy, thereby impairing mode symmetry, operational stability, and long-term drift performance [22].

Anchor loss is arguably one of the most difficult-to-predict dissipation channels in high-*Q* resonant gyroscopes, largely because it is strongly coupled to assembly conditions. Its mechanism and modeling have therefore attracted sustained research interest. Anchor-induced radiation loss is typically modeled by mainstream approaches as energy leakage via elastic waves radiates from the resonator through the anchor. These approaches commonly use a finite substrate with a perfectly matched layer (PML) to approximate the unbounded radiation boundary, thereby quantifying the energy transmitted into the substrate [23]. For anchor friction loss, microslip and the evolution of partial stick–slip at contact interfaces constitute essential physical processes. The classical Mindlin contact theory and its partial-slip solutions under varying tangential loads provide a fundamental basis for describing microslip friction dissipation and the resulting equivalent damping [24,25].

Meanwhile, manufacturing imperfections in resonators, particularly mass imbalance, can drive supports or mounting bases to participate in vibration through mode coupling [26,27]. This coupling may significantly amplify anchor-related dissipation and further induce circumferentially non-uniform damping. Existing studies have shown that an imperfect mass distribution can cause a “binding” effect between the standing wave orientation and the principal axes, which is closely related to low-order unbalanced mass harmonics [28]. Furthermore, by identifying and trimming unbalanced mass, the shell–stem coupling can be reduced and the anchor loss and damping asymmetry can be improved [29,30]. However, many prior works model friction loss or radiation loss in isolation, or discuss circumferential damping non-uniformity only under specific assembly conditions. A unified explanation remains insufficient for (i) the energy dissipation induced by externally applied tightening torque and (ii) the torque-dependent evolution of the amplitude and phase of the dominant harmonic damping component. In addition, the achievable torque range in experiments is often constrained by fixture strength and assembly limitations, making it difficult to uniquely identify multi-channel parameters from a single dataset, which further complicates model validation and engineering extrapolation.

This study uses energy dissipation as the organizing thread and takes the hemispherical resonator gyroscope (HRG) as a representative platform to develop an analysis framework for anchor loss in CVGs. First, from the viewpoint of energy channels and transfer paths, we identify the major dissipation sources that determine the quality factor and categorize anchor-related losses into two mechanisms: friction-type and radiation-type losses. Second, for friction-type anchor loss, we establish a single-cycle energy dissipation model based on the Cattaneo–Mindlin partial-slip theory; by incorporating the engineering relation between tightening torque and normal preload, we further derive a power-law scaling of torque on the mean damping, which provides a directly testable functional form for parameter identification. Third, for radiation-type anchor loss, we formulate an impedance coupling description of radiative leakage to capture the potential enhancement or saturation of energy leakage under stronger coupling, thereby offering guidance for future design extrapolation and platform extension. Finally, torque-sweep experiments are performed to fit and validate the proposed models. The results show that, within the current torque limit, the measured torque dependence of the mean damping is consistent with the frictional dissipation model, supporting a friction-dominated interpretation within the investigated torque range. In addition, we employ a harmonic characterization method that is independent of specific dissipation channels to extract and discuss the circumferential uniformity of the quality factor, and we interpret its torque dependence using a phasor-superposition framework.

## 2. Resonator Mode-Shape Analysis: Energy Dissipation Channels and Transfer Paths

### 2.1. Relationship Between the Quality Factor and Modal Energy

For a given operating mode of a resonator, the quality factor is defined as

(1) $$Q=\frac{2\pi E_{\mathrm{st}}}{\Delta W},\;Q^{-1}=\frac{\Delta W}{2\pi E_{\mathrm{st}}},$$

where $E_{\mathrm{st}}$ denotes the time-averaged stored energy of this mode over one vibration cycle, and ΔW is the total energy dissipated during the same cycle. Provided that the resonance frequency and mode shape vary only marginally, $E_{\mathrm{st}}$ can be regarded as weakly dependent on the assembly preload torque. Consequently, the influence of assembly on *Q* is mainly reflected through variations in ΔW.

### 2.2. Schematic of Energy Transfer Paths in CVGs

**Figure 1:**
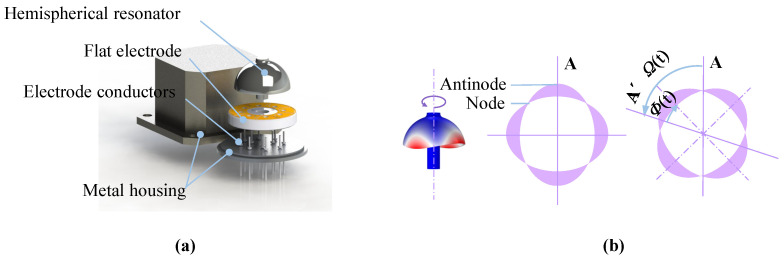
Schematic of the HRG structure. (**a**) Mechanical structure of the HRG. (**b**) Second-order vibration mode and standing-wave orientation of the HRG.

The energy transfer paths in a CVG are illustrated using an HRG as an example. As shown in Figure 1a, the mechanical structure of an HRG consists of the hemispherical resonator, planar electrodes, electrode pins, and a metallic housing, through which the device is mounted onto a base. The measurement principle is depicted in Figure 1b: The resonator vibrates in the second-order, four-antinodal flexural mode. When the gyroscope rotates, the Coriolis force induces precession of the standing wave pattern. The precession rate is sensed by the capacitive electrode array on the planar electrodes, thereby enabling the measurement of the external input angular rate.

**Figure 2:**
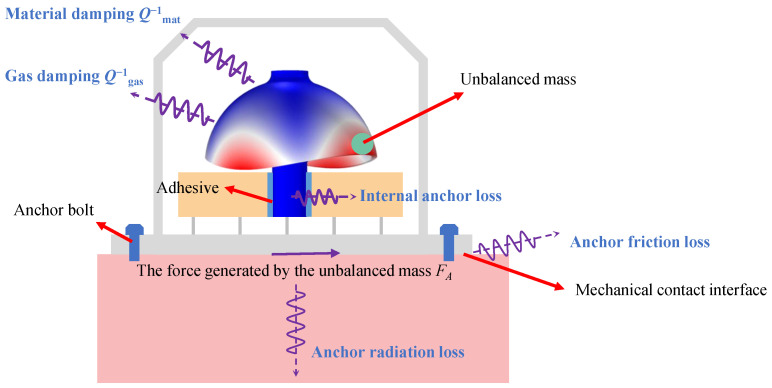
Schematic illustration of energy dissipation pathways in an HRG. Red and blue on the resonator indicate vibration displacements. The purple wavy arrows represent the energy dissipation paths. The red arrows indicate the unbalanced mass and interface features, while the purple solid arrow denotes the force $F_{A}$ generated by the unbalanced mass.

Figure 2 illustrates the major energy dissipation pathways in an HRG, including material damping, gas damping, and anchoring-related losses driven by the excitation induced by an unbalanced mass. Material loss is mainly dissipated within the resonator body (denoted by $Q_{\mathrm{mat}}^{-1}$), whereas gas damping is associated with the surrounding fluid or residual gas environment (denoted by $Q_{\mathrm{gas}}^{-1}$). Anchoring-related losses are activated by the equivalent excitation force $F_{A}$ generated by the unbalanced mass and can be further categorized into two components: (i) internal anchor loss, which is primarily associated with internal bonding structures, and (ii) mounting-induced anchor loss, arising from the coupling between the gyroscope and the supporting base through the mechanical contact interface. In general, once the device is packaged, the internal anchor loss is largely fixed by the structure and process and is difficult to alter by subsequent assembly steps. In contrast, the mounting-induced anchor loss is highly sensitive to mounting torque, contact conditions, and boundary constraints, and it typically introduces damping anisotropy, thereby degrading the symmetry and stability of the resonant modes.

This work focuses on the mounting-induced anchor loss occurring at the contact interface between the gyroscope and the base. It can be decomposed into two major mechanisms: anchor friction loss and anchor radiation loss. Anchor friction loss is caused by microslip and friction at the contact interface during vibration, while anchor radiation loss originates from elastic-wave transmission from the vibrating resonator to the base and the associated energy radiation. These mounting-related anchoring losses not only reduce the quality factor of the resonator but also introduce damping anisotropy, further impairing mode symmetry and stability. Although low-loss materials, optimized resonator design, and high-vacuum packaging can effectively mitigate the impacts of material and gas damping, anchoring loss remains challenging to control due to its strong dependence on mounting conditions, and thus it often becomes a key factor limiting further improvement of the HRG quality factor.

The total energy dissipated per cycle for a resonant mode can be decomposed as

(2) $$\Delta W=\Delta W_{\mathrm{mat}}+\Delta W_{\mathrm{gas}}+\Delta W_{\mathrm{fric}}\left(T\right)+\Delta W_{\mathrm{rad}}\left(T\right),$$

and the corresponding inverse quality factor can be written as

(3) $$Q^{-1}\left(T\right)=Q_{\mathrm{mat}}^{-1}+Q_{\mathrm{gas}}^{-1}+Q_{\mathrm{fric}}^{-1}\left(T\right)+Q_{\mathrm{rad}}^{-1}\left(T\right),$$

where $Q_{\mathrm{mat}}^{-1}$ and $Q_{\mathrm{gas}}^{-1}$ represent material internal loss and gas damping, respectively, and can be treated as constants under fixed material properties and vacuum conditions. In contrast, $Q_{\mathrm{fric}}^{-1}\left(T\right)$ and $Q_{\mathrm{rad}}^{-1}\left(T\right)$ correspond to friction-induced and elastic-wave radiation-induced anchor losses; both are strongly dependent on the assembly preload torque *T* and are the primary focus of this study.

### 2.3. A Damping Distribution Model for the Second-Order Four-Antinode Mode

**Figure 3:**
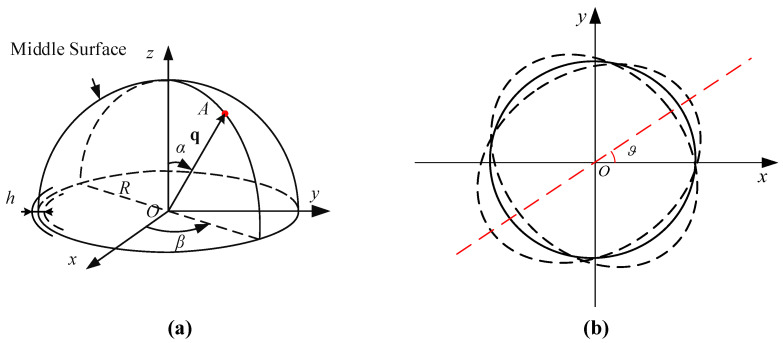
Coordinate system of the hemispherical resonator. (**a**) Three-dimensional geometric description of the hemispherical shell, where *O* is the geometric center, *x*, *y*, and *z* are the Cartesian coordinate axes, *A* is an arbitrary point on the middle surface, *R* is the middle-surface radius, *h* is the shell thickness, and the red arrow denotes the position vector $q=\vec{OA}$. α is the angle between $\vec{OA}$ and the *z*-axis, and β is the azimuth angle of the projection of $\vec{OA}$ onto the xy-plane with respect to the *x*-axis. Solid black lines indicate visible outlines and axes, while black dashed lines indicate hidden contours. (**b**) Top view of the resonator showing the standing-wave orientation, where the black solid circle denotes the reference contour, the black dashed curves denote the deformed contours at two opposite vibration phases, the red dashed line denotes the antinode direction, and ϑ is the corresponding azimuth angle measured from the *x*-axis.

The hemispherical resonator can be equivalently modeled as a hemispherical shell, as shown in Figure 3. Let the geometric center of the resonator be the origin *O* of the Cartesian coordinate system *O*–xyz. The position of an arbitrary point *A* on the middle surface can be described using spherical parameters, where *R* is the middle-surface radius, α is the angle between the vector $\vec{OA}$ and the *z*-axis, and β is the azimuth angle of the projection of $\vec{OA}$ onto the xy-plane with respect to the *x*-axis. Here, ϑ denotes the azimuth orientation of the standing wave antinode direction relative to the *x*-axis.

When operating in the second-order, four-antinodal flexural mode, the dominant displacement component at geometric azimuth β can be expressed as

(4) $$u\left(\beta ,\vartheta \right)=u_{0}\cos2\left(\beta -\vartheta \right).$$

The local damping can be regarded as being distributed along the circumferential direction β and represented by infinitesimal resonator elements. The local energy dissipation is proportional to the local strain energy density, which, for the present mode, is approximated to scale with the squared displacement. Accordingly, the normalized energy weighting at azimuth β is approximated as

(5) $$w\left(\beta ,\vartheta \right)\approx \frac{u^{2}\left(\beta ,\vartheta \right)}{u_{0}^{2}}=\cos^{2}\;2\left(\beta -\vartheta \right)=\frac{1}{2}\left[1+\cos4(\beta -\vartheta )\right].$$

It can be seen that even if the local damping itself contains no fourth-order term, the modal energy weighting of the second-order mode inherently introduces a fourth-harmonic factor through $\cos\left(4(\beta -\vartheta )\right)$.

Let d(β) denote the equivalent damping (or the loss per unit strain energy) at circumferential position β. For a given wave orientation ϑ, the modal equivalent loss can be written as

(6) $$D\left(\vartheta \right)\;=\;\int_{0}^{2\pi }d\left(\beta \right)\;w\left(\beta ,\vartheta \right)\;d\beta =\frac{1}{2}\int_{0}^{2\pi }d\left(\beta \right)\;d\beta +\frac{1}{2}\int_{0}^{2\pi }d\left(\beta \right)\cos4\left(\beta -\vartheta \right)\;d\beta .$$

Expanding the local damping in a Fourier series and retaining the 0th and 4th terms that couple most strongly with the second-order mode yield

(7) $$d\left(\beta \right)\approx d_{0}+d_{4}\cos\left[4(\beta -\beta_{d})\right],$$

where $d_{0}$ is the mean damping level, $d_{4}$ is the amplitude of the fourth-harmonic component, and $\beta_{d}$ is the principal-axis azimuth of the damping harmonic. Substituting into the above expression and using orthogonality leads to the modal equivalent loss

(8) $$D\left(\vartheta \right)\approx D_{0}+D_{4}\cos\left[4(\vartheta -\beta_{d})\right],$$

where $D_{0}$ is associated with the circumferentially averaged damping and $D_{4}$ is proportional to $d_{4}$. Accordingly, the azimuthal dependence of the quality factor can be expressed as

(9) $$\frac{1}{Q(\vartheta )}=\frac{1}{Q_{0}}+\Delta D\left(\vartheta \right)\approx \frac{1}{Q_{0}}+D_{4}\cos\left[4(\vartheta -\varphi_{d})\right],$$

η(ϑ)=1/Q(ϑ) exhibits a dominant fourth-harmonic dependence on 4ϑ, with the amplitude determined by $D_{4}$ and the principal orientation determined by $\varphi_{d}$.

In the ideal axisymmetric case with isotropic damping, $d_{4}=0$ and thus $D_{4}=0$; Equation (9) reduces to

(10) $$\frac{1}{Q(\vartheta )}\equiv \frac{1}{Q_{0}},$$

indicating that the quality factor is independent of azimuth.

In practical structures, the anchoring pre-stress and assembly constraints can induce a non-uniform circumferential distribution of local damping d(β), resulting in a finite fourth-harmonic component $d_{4}$ and hence $D_{4}\neq 0$. When the mounting condition is altered—for example, by changing the screw torque, clamping configuration, or the degree of asymmetry—the following effects may occur:

- The strength of damping anisotropy changes, i.e., $d_{4}$ varies, which directly modifies the fourth-harmonic amplitude $D_{4}$ in Q(ϑ);
- The principal direction of the damping distribution changes, i.e., $\varphi_{d}$ varies, which causes an overall azimuthal rotation of the fourth-harmonic pattern of Q(ϑ).

Therefore, for an HRG operating in the second-order, four-antinodal mode, damping non-uniformity is primarily manifested in $Q^{-1}$ as a fourth-harmonic component,

(11) $$\Delta \;\left(Q^{-1}\right)\left(\vartheta \right)=D_{4}\cos\left[4(\vartheta -\varphi_{d})\right].$$

Variations in the assembly and the resulting anchoring pre-stress distribution predominantly affect the damping anisotropy and the azimuthal uniformity of *Q* by changing the amplitude $D_{4}$ and the orientation $\varphi_{d}$ of this fourth-harmonic term.

## 3. Modeling of Energy Dissipation Due to Anchor Friction

At the microscopic level, the anchoring interface can be viewed as an ensemble of local micro-contact asperities that collectively sustain the normal preload and accommodate tangential microslip. For the *i*-th micro-contact, the contact can be approximated as a Hertzian circular contact subjected to a constant normal load $F_{N,i}$ and a harmonic tangential load,

(12) $$F_{T,i}\left(t\right)=F_{A,i}\sin\omega t,\;F_{A,i}<\mu F_{N,i},$$

where $F_{A,i}$ is the tangential force amplitude at this micro-contact and μ is the Coulomb friction coefficient.

When $0<\lambda_{i}<1$ with $\lambda_{i}=F_{A,i}/\left(\mu F_{N,i}\right)$, the interface operates in the Cattaneo–Mindlin partial-slip regime. In this regime, the tangential force–displacement relation exhibits hysteresis, and the frictional energy dissipated per cycle equals the area enclosed by the hysteresis loop. For a Hertzian circular contact, Mindlin’s classical result provides a closed-form expression for the per-cycle energy dissipation [31]:

(13) $$|\Delta W_{i}|=\frac{9\mu^{2}F_{N,i}^{2}\left(2-\nu \right)}{5Ga_{i}}\;\Psi \left(\lambda_{i}\right),\;\lambda_{i}=\frac{F_{A,i}}{\mu F_{N,i}},$$

where

(14) $$\Psi \left(\lambda_{i}\right)=1-{\left(1-\lambda_{i}\right)}^{\frac{5}{3}}-\frac{5}{6}\lambda_{i}\left[1+{\left(1-\lambda_{i}\right)}^{\frac{2}{3}}\right],\;0<\lambda_{i}<1,$$

and *G* and ν are the shear modulus and Poisson’s ratio, respectively, while $a_{i}$ is the effective contact radius.

To derive an identifiable scaling law, we consider the small-amplitude partial-slip regime ($\lambda_{i}\ll 1$). Expanding $\Psi (\lambda_{i})$ and neglecting higher-order terms yields [32]

(15) $$\Psi \left(\lambda_{i}\right)\approx \alpha_{3}\lambda_{i}^{3},\;\alpha_{3}=\frac{5}{162}.$$

Moreover, for a Hertzian circular contact, $a_{i}\propto F_{N,i}^{1/3}$, implying $F_{N,i}^{2}/a_{i}\propto F_{N,i}^{5/3}$. Substituting Equation (15) and $\lambda_{i}=F_{A,i}/\left(\mu F_{N,i}\right)$ into Equation (13) gives the leading-order scaling for a single micro-contact:

(16) $$|\Delta W_{i}|\propto \mu^{2}F_{N,i}^{5/3}{\left(\frac{F_{A,i}}{\mu F_{N,i}}\right)}^{3}=c_{i}\;F_{A,i}^{3}\;F_{N,i}^{-4/3},$$

where $c_{i}$ collects constants associated with material properties, contact geometry, and $\alpha_{3}$.

The total frictional energy dissipation at the anchoring interface is the sum over all micro-contacts:

(17) $$\Delta W_{\mathrm{fric}}=\sum_{i\in C}\Delta W_{i}.$$

If the load sharing among micro-contacts satisfies $F_{N,i}=w_{i}F_{N}$, and the second-order dependence of the tangential excitation amplitude $F_{A,i}$ on the tightening torque can be absorbed into an effective coefficient, Equation (17) retains the same dominant exponent as the single-contact scaling:

(18) $$\Delta W_{\mathrm{fric}}\left(F_{N}\right)\approx C_{\mathrm{fric}}\;F_{N}^{-p_{\mathrm{fric}}},\;p_{\mathrm{fric}}=\frac{4}{3},$$

where $C_{\mathrm{fric}}$ incorporates coefficients related to contact rearrangement, such as $\sum_{i}c_{i}F_{A,i}^{3}w_{i}^{-4/3}$.

In practical anchoring structures, the normal preload $F_{N}$ is typically introduced by an applied tightening torque *T*. Within the elastic tightening range, an approximately linear torque–preload relationship is commonly assumed in engineering practice and can be expressed as

(19) $$F_{N}\approx \kappa T,$$

where κ is an effective torque coefficient determined by the thread geometry, nominal diameter, contact configuration, and friction conditions. Such a linear approximation is widely adopted in fastener mechanics and preload characterization under controlled tightening conditions [33,34].

Substituting Equation (19) into Equation (18) yields the torque scaling of the frictional anchor dissipation:

(20) $$\Delta W_{\mathrm{fric}}\left(T\right)=C_{\mathrm{fric}}\kappa^{-p_{\mathrm{fric}}}T^{-p_{\mathrm{fric}}}.$$

Furthermore, the contribution of the friction channel to the damping factor (or the inverse quality factor) is given by the ratio between the per-cycle dissipated energy and the modal stored energy $E_{\mathrm{st}}$:

(21) $$Q_{\mathrm{fric}}^{-1}\left(T\right)=\frac{\Delta W_{\mathrm{fric}}\left(T\right)}{2\pi E_{\mathrm{st}}}=A\;T^{-p_{\mathrm{fric}}},$$

where $A=C_{\mathrm{fric}}\kappa^{-p_{\mathrm{fric}}}/\left(2\pi E_{\mathrm{st}}\right)$ is a constant.

## 4. Modeling of Elastic-Wave Radiation Anchor Loss

In addition to anchor friction, the resonator may also lose energy by radiating elastic waves through the anchoring structure into the base. To formulate a unified model that is consistent with the preceding sections, we adopt an impedance coupling description based on an “equivalent anchoring stiffness + base drive-point mobility” representation.

Let the operating angular frequency be $\omega_{0}$. The effective displacement amplitude of the resonant mode at the anchoring location is denoted by $x_{r}=\alpha q$, where α is determined by the mode shape and *q* is the generalized coordinate amplitude. The coupling strength between the resonator and the base is characterized by an equivalent anchoring stiffness $K_{a}\left(T\right)$, which depends on the tightening torque *T*. The drive-point velocity mobility of the base at the anchoring point is defined as

(22) $$Y_{b}\left(\omega \right)=\frac{V_{b}}{F_{A}},$$

where $F_{A}$ is the reaction-force amplitude transmitted to the base and $V_{b}$ is the corresponding velocity amplitude at the base point.

The relative displacement across the anchor satisfies $F_{A}=K_{a}\left(T\right)\left(x_{r}-x_{b}\right)$, and $x_{b}=V_{b}/\left(j\omega_{0}\right)=Y_{b}\left(\omega_{0}\right)F_{A}/\left(j\omega_{0}\right)$. Therefore, the transmitted reaction force can be expressed as

(23) $$F_{A}=\frac{K_{a}\left(T\right)}{1+\frac{K_{a}\left(T\right)}{j\omega_{0}}Y_{b}\left(\omega_{0}\right)}\;x_{r}=\frac{K_{a}\left(T\right)\alpha }{1+\frac{K_{a}\left(T\right)}{j\omega_{0}}Y_{b}\left(\omega_{0}\right)}\;q.$$

When the base can be approximated as a semi-infinite medium, or when the effective loss is dominated by radiation, the cycle-averaged radiated power through the anchor can be written as

(24) $${\bar{P}}_{\mathrm{rad}}\left(T\right)=\frac{1}{2}ℜ\;\left\{F_{A}\;V_{b}^{*}\right\}=\frac{1}{2}{\left|F_{A}\right|}^{2}\;ℜ\;\left\{Y_{b}\left(\omega_{0}\right)\right\}.$$

Using the modal time-averaged stored energy $E_{\mathrm{st}}=\frac{1}{2}M_{\mathrm{eff}}\omega_{0}^{2}{\left|q\right|}^{2}$ and the definition $Q^{-1}=\Delta W/\left(2\pi E_{\mathrm{st}}\right)=\bar{P}/\left(\omega_{0}E_{\mathrm{st}}\right)$, Equation (24) yields the radiation-induced anchor-loss contribution

(25) $$Q_{\mathrm{rad}}^{-1}\left(T\right)=\frac{{\bar{P}}_{\mathrm{rad}}\left(T\right)}{\omega_{0}E_{\mathrm{st}}}=C_{\mathrm{rad}}\;ℜ\;\left\{Y_{b}\left(\omega_{0}\right)\right\}\;{\left|\frac{K_{a}\left(T\right)}{1+\frac{K_{a}\left(T\right)}{j\omega_{0}}Y_{b}\left(\omega_{0}\right)}\right|}^{2},$$

where $C_{\mathrm{rad}}=\alpha^{2}/\left(M_{\mathrm{eff}}\omega_{0}^{3}\right)$ is a mode-normalization constant.

In general, increasing the tightening torque *T* enhances the equivalent anchoring stiffness $K_{a}\left(T\right)$ due to enlarged contact area, higher clamping pressure, and strengthened structural constraint. To provide a generic scaling description, $K_{a}\left(T\right)$ can be approximated by a power law:

(26) $$K_{a}\left(T\right)=K_{a0}T^{p_{a}},\;p_{a}>0.$$

Equation (25) directly leads to two limiting regimes:

- Weak coupling/compliant anchoring ($K_{a}\left|Y_{b}\right|/\omega_{0}\ll 1$):
  (27) $$Q_{\mathrm{rad}}^{-1}\left(T\right)\approx C_{\mathrm{rad}}ℜ\;\left\{Y_{b}\right\}\;{\left|K_{a}\left(T\right)\right|}^{2}\propto T^{2p_{a}};$$
- Strong coupling/stiff anchoring ($K_{a}\left|Y_{b}\right|/\omega_{0}\gg 1$):
  (28) $$Q_{\mathrm{rad}}^{-1}\left(T\right)\approx C_{\mathrm{rad}}ℜ\;\left\{Y_{b}\right\}{\left|\frac{j\omega_{0}}{Y_{b}\left(\omega_{0}\right)}\right|}^{2}=C_{\mathrm{rad}}\;\omega_{0}^{2}\;\frac{ℜ\;\{Y_{b}\}}{|Y_{b}{|}^{2}},$$
  in which case the radiation loss approaches saturation, and further increasing the anchoring stiffness has a limited effect on the radiated loss.

## 5. Results and Discussion

In the preceding sections, anchoring-related losses were modeled in a channel-wise manner, with energy dissipation as the organizing principle. For the friction channel, a per-cycle dissipation model was established based on Cattaneo–Mindlin partial-slip contact, and, together with Hertz contact scaling and preload relations, a power-law dependence of the mean damping on tightening torque was obtained. For the radiation channel, an impedance coupling description was formulated to represent elastic-wave energy leakage into the base, capturing the possible enhancement and saturation trends under stronger coupling. With these models, the torque-dependent inverse quality factor can be written in a unified form as

(29) $$Q^{-1}\left(T\right)=Q_{0}^{-1}+Q_{\mathrm{fric}}^{-1}\left(T\right)+Q_{\mathrm{rad}}^{-1}\left(T\right),$$

where $Q_{0}^{-1}$ collects background losses that are weakly dependent on torque, while $Q_{\mathrm{fric}}^{-1}\left(T\right)$ and $Q_{\mathrm{rad}}^{-1}\left(T\right)$ denote the torque-dependent contributions from friction-type and radiation-type anchor losses, respectively. This section uses torque-sweep experiments to identify the torque scaling of the mean term in Equation (29), thereby determining the dominant dissipation mechanism within the practical assembly range. In parallel, the circumferential uniformity of damping is extracted and analyzed to explain how the spatial distribution of *Q* evolves with tightening torque. The experimental setup and procedure are described first, followed by the fitting results and discussion.

**Figure 4:**
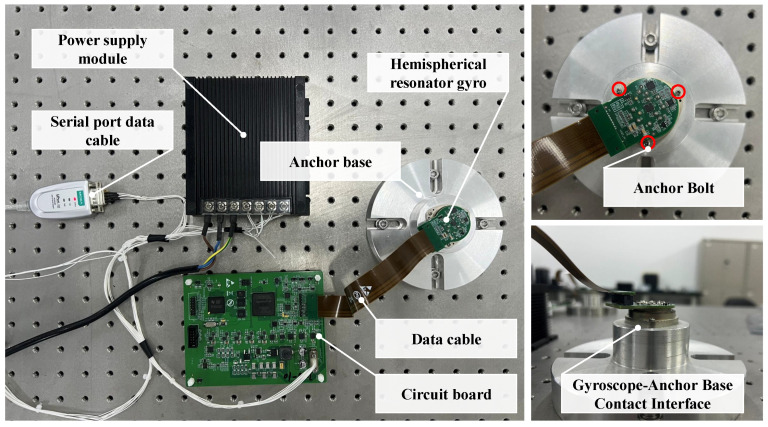
Experimental platform.

Figure 4 shows the experimental platform, which consists of the HRG under test, a rigid base, and the excitation-and-measurement electronics. The HRG is mounted to the rigid base using three bolts, and the base is fixed on the test bench. The anchoring preload is controlled by adjusting the bolt tightening torque *T*. The control electronics provide resonator excitation and signal acquisition for energy decay measurements.

**Figure 5:**
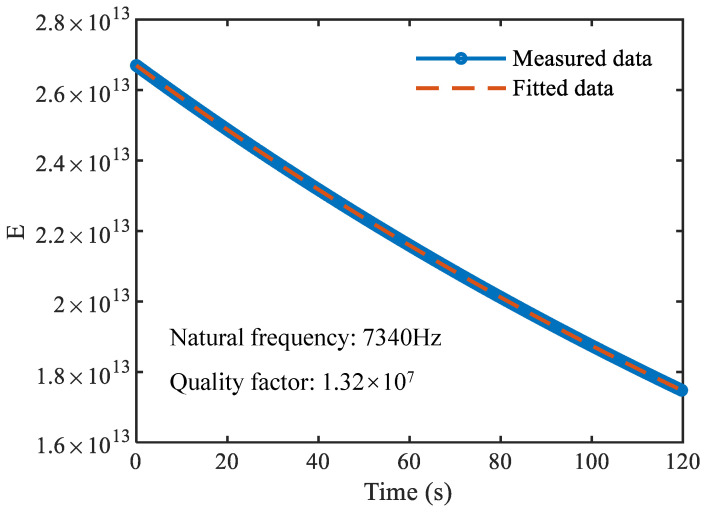
Energy decay curve at a standing wave orientation of $0^{{}^{\circ}}$.

A full-angle control scheme is employed to evaluate the quality factor at different standing wave orientations. For a given torque level, the standing wave is first actively driven to a prescribed azimuth ϑ. Next, the amplitude-control loop is opened to allow free decay, and the modal energy E(t) is reconstructed from the measured signals. The quality factor at this orientation is then obtained by exponential fitting,

(30) $$E\left(t\right)=E_{0}\exp\;\left(-\frac{\omega_{0}}{Q}t\right),$$

where $E_{0}$ is the initial energy and $\omega_{0}$ is the resonance frequency. Figure 5 provides an example of the energy decay curve at $\vartheta =0^{{}^{\circ}}$, from which a quality factor of $Q=1.32\times 10^{7}$ is obtained for the tested device.

For each tightening torque *T*, the quality factor Q(T,ϑ) is measured over standing wave orientations $\vartheta \in [0^{{}^{\circ}},180^{{}^{\circ}}]$ with a step size of $10^{{}^{\circ}}$. To remain consistent with the energy-loss modeling in the previous sections, we use the damping factor (inverse quality factor),

(31) $$\eta \left(T,\vartheta \right)=\frac{1}{Q(T,\vartheta )},$$

as the fitting target. For each torque level, we extract the mean term $D_{0}\left(T\right)$ and the dominant fourth-harmonic component characterized by its amplitude $D_{4}\left(T\right)$ and phase $\phi_{d}\left(T\right)$, and then analyze their dependence on tightening torque.

### 5.1. Fourth-Harmonic Fitting of the Circumferential Distribution

**Figure 6:**
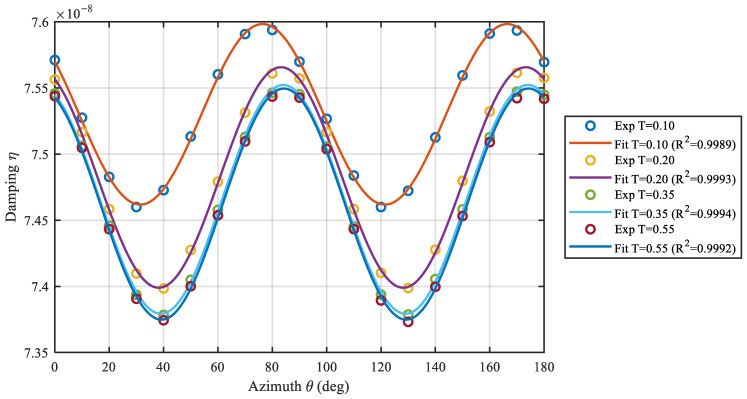
Fourth-harmonic fitting of the circumferential distribution of η(T,ϑ)=1/Q(T,ϑ).

Due to the circumferential symmetry of the second-order four-antinodal mode, η(T,ϑ) exhibits a pronounced periodicity with 4ϑ. Accordingly, a fourth-harmonic model is used to fit the circumferential distribution for each torque *T* via least squares. Table 1 lists the fitted parameters $D_{0}$, $D_{4}$, ε, $\phi_{d}$, and the goodness-of-fit $R^{2}$. For all torque levels, $R^{2}$ remains close to unity, indicating that the circumferential variation in η(T,ϑ) is consistently captured by the fourth-harmonic model under the present experimental conditions. This observation supports the fourth-harmonic distribution implied by the energy-dissipation analysis. Representative fits are shown in Figure 6.

### 5.2. Mean Damping $D_{0}\left(T\right)$ Versus Tightening Torque

As seen in Table 1, the mean damping $D_{0}\left(T\right)$ decreases monotonically with increasing *T* and gradually approaches saturation. When *T* increases from 0.10 to 0.55, $D_{0}$ decreases from $7.53\times 10^{-8}$ to $7.462\times 10^{-8}$, corresponding to a relative change of about 0.9%. This trend is consistent with the friction-channel picture in the partial-slip regime: higher preload reduces microslip and thus lowers frictional dissipation.

For engineering use, we describe the torque dependence of $D_{0}\left(T\right)$ using the general power-law form

(32) $$D_{0}\left(T\right)=D_{\mathrm{base}}+A\;T^{-p},$$

where $D_{\mathrm{base}}$ denotes an effective torque-insensitive background term, and $A\;T^{-p}$ represents the assembly-dependent contribution.

To assess whether the exponent can be inferred directly from the data, we first performed an unconstrained fit of Equation (32), in which *p* was treated as an adjustable parameter. The resulting fit yields $D_{\mathrm{base}}=7.4539\times 10^{-8}$, $A=3.04\times 10^{-11}$, p=1.3864, with a 95% confidence interval of p∈[0.9242,1.8485].

The corresponding fitting metrics are $R^{2}=0.98756$, $\mathrm{RMSE}=2.217\times 10^{-11}$, $\max|e|=5.651\times 10^{-11}$. Importantly, the confidence interval includes the theoretical value p=4/3, indicating that the experimental data are compatible with the partial-slip friction scaling.

Within the partial-slip friction framework, the friction channel suggests a power-law dependence with exponent p=4/3 with respect to preload. To preserve this physical interpretability, we additionally considered the constrained form

(33) $$D_{0}\left(T\right)=D_{\mathrm{base}}+A\;T^{-4/3}.$$

Because Equation (33) is linear in $\left(D_{\mathrm{base}},A\right)$, a linear least-squares estimate can be performed using $x=T^{-4/3}$. The resulting parameters are $D_{\mathrm{base}}=7.4530\times 10^{-8}$, $A=3.43\times 10^{-11}$, with fitting metrics $R^{2}=0.98615$, $\mathrm{RMSE}=2.339\times 10^{-11}$, $\max|e|=5.668\times 10^{-11}$.

For comparison, we also examined an alternative monotonic exponential form

(34) $$D_{0}\left(T\right)=D_{\infty }+B\;e^{-cT},$$

which gave $R^{2}=0.93628$, $\mathrm{RMSE}=5.018\times 10^{-11}$, $\max|e|=1.421\times 10^{-10}$. Compared with the two power-law fits, the exponential model shows significantly poorer agreement with the data.

**Figure 7:**
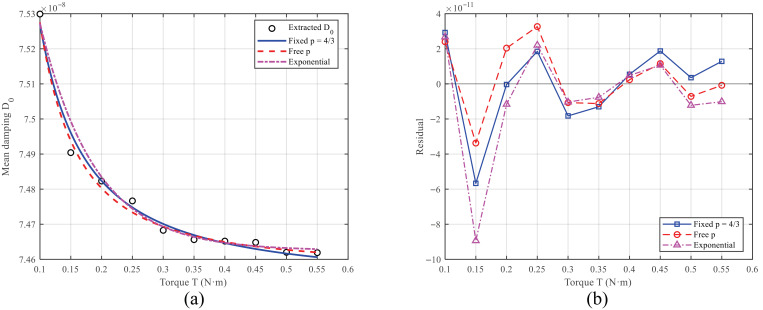
Comparison of fitting models for the mean damping $D_{0}\left(T\right)$: (**a**) extracted $D_{0}\left(T\right)$ together with the constrained power-law fit (p=4/3), the unconstrained power-law fit, and the exponential fit; (**b**) corresponding fitting residuals.

Figure 7a compares the extracted $D_{0}\left(T\right)$ with the constrained power-law fit, the unconstrained power-law fit, and the exponential fit, while Figure 7b shows the corresponding residuals. The unconstrained power-law fit provides a slightly better $R^{2}$ and RMSE than the fixed-*p* fit, but the improvement is modest.

Therefore, the present data do not provide a strong independent identification of the exponent. Rather, the fitting comparison shows that the measured trend is well described by a power-law model and is compatible with the partial-slip friction picture. In this sense, the constrained fit with p=4/3 is retained as a physically interpretable theory-consistency fit, while the unconstrained fit demonstrates that the experimental data do not contradict the theoretically predicted exponent.

Results further indicate that $D_{\mathrm{base}}$ is the dominant contribution, whereas the torque-dependent term serves as a secondary correction. This suggests that, in the tested torque range, the assembly-induced change in loss is measurable but not dominant. Accordingly, although the radiation-loss model can predict an increasing damping trend under strong coupling, the measured $D_{0}\left(T\right)$ decreases monotonically and is more consistent with the friction-loss trend in the present regime. We therefore interpret the current data as supporting a friction-dominated regime within the tested torque range, while discussing the radiation channel mainly as a complementary mechanism for extrapolation rather than as an independently validated dominant dissipation source.

### 5.3. Fourth-Harmonic Amplitude $D_{4}\left(T\right)$ Versus Tightening Torque

**Figure 8:**
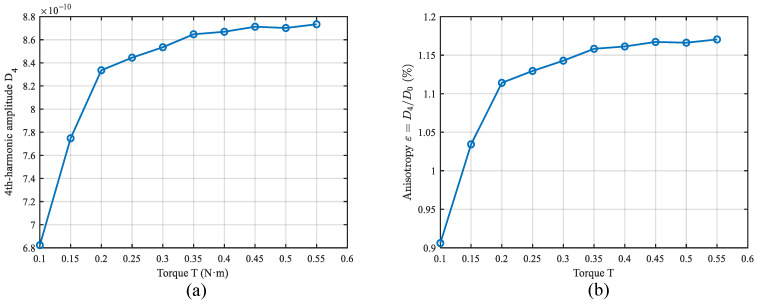
Evolution of (**a**) the fourth-harmonic amplitude $D_{4}\left(T\right)$, and (**b**) the anisotropy index ε(T) with tightening torque.

As shown in Figure 8a, the fourth-harmonic amplitude $D_{4}\left(T\right)$ exhibits a “rapid increase followed by saturation” with increasing torque. It increases from $6.82\times 10^{-10}$ to approximately $8.73\times 10^{-10}$, about a 28% increase, while the anisotropy index ε(T) rises from 0.906% to 1.170%. This indicates that, although the overall dissipation decreases slightly with torque, the circumferentially non-uniform component becomes more pronounced in relative terms.

A single-source fourth-harmonic term is not sufficient to explain both the increase in $D_{4}$ and the marked rotation of $\phi_{d}$. To interpret these coupled trends, we introduce a unified picture based on phasor superposition of multiple fourth-harmonic contributions.

**Figure 9:**
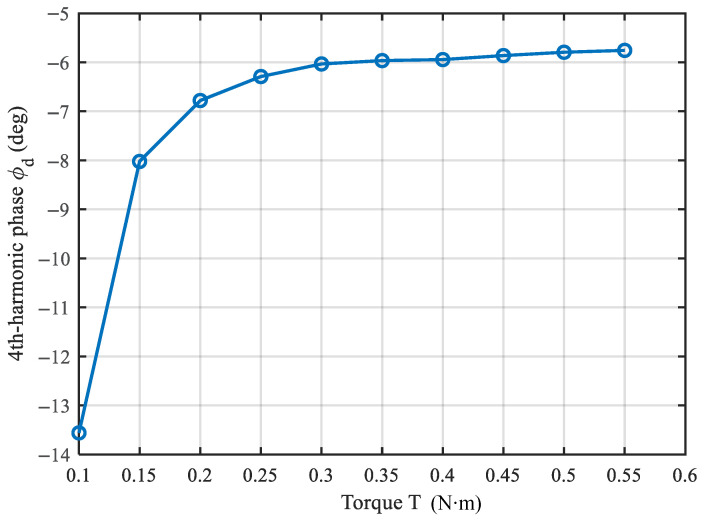
Evolution of the fourth-harmonic phase $\phi_{d}\left(T\right)$ with tightening torque.

Figure 9 shows that $\phi_{d}\left(T\right)$ rotates and gradually converges with increasing torque: $\phi_{d}$ changes monotonically from $-13.56^{{}^{\circ}}$ to $-5.76^{{}^{\circ}}$, with most of the change occurring at low torques (T≤0.20). For T≥0.35, the phase variation becomes small, suggesting that the anchoring interface approaches a more stable contact state.

To describe the coupled evolution of $D_{4}\left(T\right)$ and $\phi_{d}\left(T\right)$ in a compact form, we define a complex fourth-harmonic phasor

(35) $${\tilde{D}}_{4}\left(T\right)=D_{4c}\left(T\right)-jD_{4s}\left(T\right),\;\eta \left(T,\vartheta \right)\approx D_{0}\left(T\right)+ℜ\left\{{\tilde{D}}_{4}\left(T\right)e^{j4\vartheta }\right\}.$$

Then,

(36) $$D_{4}\left(T\right)=\left|{\tilde{D}}_{4}\left(T\right)\right|,\;\phi_{d}\left(T\right)=\frac{1}{4}\arg\;\left({\tilde{D}}_{4}\left(T\right)\right).$$

We further decompose the total phasor as

(37) $${\tilde{D}}_{4}\left(T\right)={\tilde{D}}_{4,\mathrm{mass}}+{\tilde{D}}_{4,\mathrm{anch}}\left(T\right),$$

where:

- ${\tilde{D}}_{4,\mathrm{mass}}$ represents a torque-insensitive fourth-harmonic term associated with mass imperfections. Under the ideal assumption that the anchoring is circumferentially uniform and that the transfer weighting does not vary with torque, its phase is primarily determined by the defect orientation and is approximately independent of *T*.
- ${\tilde{D}}_{4,\mathrm{anch}}\left(T\right)$ represents an additional fourth-harmonic term introduced by anchoring non-uniformity, contact-state rearrangement, and potentially torque-dependent radiation coupling; both its amplitude and phase may vary with *T*.

The phase-convergence behavior suggests that, beyond a certain torque level, the influence of ${\tilde{D}}_{4,\mathrm{anch}}\left(T\right)$ becomes weaker, and the overall fourth harmonic is increasingly governed by ${\tilde{D}}_{4,\mathrm{mass}}$. Using the phasor average in a high-torque interval, e.g., *T* = 0.45–0.55 N·m, as an approximation of ${\tilde{D}}_{4,\mathrm{mass}}$, we estimate $\phi_{\mathrm{mass}}\approx -5.8^{{}^{\circ}}$, which agrees with the converged value observed for $\phi_{d}\left(T\right)$. At low torque, ${\tilde{D}}_{4,\mathrm{anch}}\left(T\right)$ is comparable in magnitude to ${\tilde{D}}_{4,\mathrm{mass}}$ but oriented differently, leading to a noticeable phasor rotation and partial cancellation. This explains why $D_{4}$ is smaller and $\phi_{d}$ deviates more from the converged phase at low torques. As torque increases, the anchoring contact state progressively stabilizes, so that the anchoring-related fourth-harmonic contribution varies less with torque. Consequently, ${\tilde{D}}_{4,\mathrm{anch}}\left(T\right)$ decreases or approaches a constant, causing $D_{4}\left(T\right)$ to increase and then saturate, while $\phi_{d}\left(T\right)$ converges toward $\phi_{\mathrm{mass}}$.

## 6. Conclusions

This work develops a torque-dependent framework for analyzing anchor loss in CVGs, with energy dissipation as the central theme, and validates the key implications using torque-sweep experiments on an HRG. The main conclusions are as follows:

(1) A per-cycle dissipation model for friction-type anchor loss is derived from the Cattaneo–Mindlin partial-slip theory. Combined with Hertzian contact scaling and preload relations, the model yields a power-law form for the mean damping as a function of tightening torque, enabling direct parameter identification from experimental data.
(2) The experimentally extracted mean damping decreases slightly with increasing torque and is accurately captured by the proposed power-law scaling within the accessible torque range, indicating that friction-type anchor loss dominates the torque-dependent dissipation in this regime. The radiation-loss model is retained primarily for design extrapolation under stronger coupling conditions and extended torque ranges, rather than as the dominant mechanism identified in the present tests.
(3) The circumferential variation of damping is consistently dominated by a fourth-harmonic component, allowing a compact harmonic characterization of quality-factor non-uniformity. A phasor-superposition interpretation explains the coupled torque-dependent evolution of the fourth-harmonic amplitude and phase, suggesting that preload-induced contact-state stabilization and rearrangement are key drivers of the observed changes in anisotropy.

These results provide quantitative scaling guidance for mean loss and a practical framework for uniformity analysis, offering theoretical and experimental support for anchoring-structure design and assembly-process optimization in resonant gyroscopes. Future work may extend the torque range and increase coupling strength to further examine a possible transition toward radiation-dominated loss and to refine unified identification methods for multi-channel dissipation.

## Figures and Tables

**Table 1:** Mean damping and fourth-harmonic parameters under different tightening torques.

T(N·m)	$D_{0}\;\left(\times 10^{-8}\right)$	$D_{4}\;\left(\times 10^{-10}\right)$	$\varepsilon =D_{4}/D_{0}\;\left(\%\right)$	$\phi_{d}\;{(}^{{}^{\circ}})$	$R^{2}$
0.10	7.5300	6.823	0.906	−13.560	0.998856
0.15	7.4905	7.747	1.034	−8.023	0.999215
0.20	7.4823	8.336	1.114	−6.779	0.999297
0.25	7.4769	8.445	1.129	−6.290	0.999202
0.30	7.4685	8.535	1.143	−6.033	0.999164
0.35	7.4658	8.647	1.158	−5.965	0.999396
0.40	7.4652	8.669	1.161	−5.944	0.999372
0.45	7.4651	8.713	1.167	−5.862	0.999188
0.50	7.4621	8.702	1.166	−5.794	0.999328
0.55	7.4622	8.734	1.170	−5.756	0.999181

## Data Availability

Data are available from the corresponding author upon reasonable request.
